# The rhythmic mind: brain functions of percussionists in improvisation

**DOI:** 10.3389/fnhum.2024.1418727

**Published:** 2024-07-15

**Authors:** Yin-Chun Liao, Ching-Ju Yang, Hsin-Yen Yu, Chiu-Jung Huang, Tzu-Yi Hong, Wei-Chi Li, Li-Fen Chen, Jen-Chuen Hsieh

**Affiliations:** ^1^Institute of Brain Science, National Yang Ming Chiao Tung University, Taipei, Taiwan; ^2^Integrated Brain Research Unit, Department of Medical Research, Taipei Veterans General Hospital, Taipei, Taiwan; ^3^Department of Biological Science and Technology, College of Biological Science and Technology, National Yang Ming Chiao Tung University, Hsinchu, Taiwan; ^4^Graduate Institute of Arts and Humanities Education, Taipei National University of the Arts, Taipei, Taiwan; ^5^Center for Intelligent Drug Systems and Smart Bio-devices, National Yang Ming Chiao Tung University, Hsinchu, Taiwan; ^6^Brain Research Center, National Yang Ming Chiao Tung University, Taipei, Taiwan

**Keywords:** percussionists, network for musical rhythm, structural improvisation, free improvisation, functional magnetic resonance imaging

## Abstract

**Introduction:**

Percussionists stand out for their expertise in rhythm, with the network for musical rhythm (NMR) serving a vital neurological function in their improvisation, which is deeply rooted in comprehensive musical knowledge. Our research examines the central representations of various improvisation tactics used by percussionists and investigates the interactions between the NMR and other relevant neural networks.

**Methods:**

Twenty-five percussionists participated in functional magnetic resonance imaging (fMRI) sessions, which included two cognitive strategies of improvisation. Structural improvisation (SIMP) emphasized rhythmic patterns, while free improvisation (FIMP) focused on musical spontaneity. Sight-reading scenario served as the reference condition. Paired *t*-tests were utilized for comparative analyses.

**Results:**

The findings revealed a dynamic interplay characterized by increased activity in the executive control network and NMR, along with decreased activity in the default mode network during SIMP. During FIMP, heightened activity was observed in the executive control network, NMR, limbic, and memory systems. In both SIMP vs. sight-reading and FIMP vs. sight-reading comparisons, the visual network’s activity decreased, a trend also observed in the comparative analysis of FIMP vs. SIMP.

**Discussion:**

In SIMP, percussionists leverage external rhythmic signals, resulting in heightened NMR and ECN activity and reduced DMN activity. In contrast, FIMP is characterized by a rise in activity within the NMR, ECN, limbic system, memory system, and reward system, underscoring the vital roles of motivation and memory in the rapid production of spontaneous musical ideas within set frameworks. The diminished activity in the visual network during FIMP compared to SIMP suggests less reliance on visual stimuli in FIMP. These findings suggest that various improvisational tactics may engage different neural pathways.

## 1 Introduction

Musical improvisation manifests musical creativity, embodying a spontaneous and unplanned artistic expression that incorporates performers’ novel and innovative musical concepts (Biasutti and Frezza, 2009; Biasutti, 2015). These musical ideas evolve from the extension of performers’ prior musical experiences, encompassing emotions, cognition, perceptual references, the knowledge base, and musical memories (Biasutti, 2015, 2017; Loui, 2018). Engaging in improvisation demands profound instrumental and motor skills and a deep understanding of musical knowledge (Biasutti, 2015, 2017). The improvisational framework categorizes creativity into three distinct levels: the computational level, which embodies systematic goals for successful improvisation; the algorithmic level, delineating the cognitive processes and transformations devised to attain these goals; and the implementational level, which represents the neural substrate for executing the requisite cognitive processes (Loui, 2018). Percussionists enhance their music with various qualities, notably mastering complex rhythms, a key indicator of their expertise. Through extensive professional training, percussionists may achieve proficiency across three levels.

Improvisation spans a range of styles, such as jazz, hip-hop, disco beat, Motown groove, blues, and African drumming, where rhythm is a key component across all forms. Improvisation involves extensive utilization of cognitive processes and transitions (Beaty, 2015; Loui, 2018; Faber and McIntosh, 2019). Previous studies on musical improvisation have disclosed the engagement of many neural networks. The network for musical rhythm (NMR), the fundamental neural network of percussion art, is crucial for percussion interpretation, improvisation strategies, and performance (Chen et al., 2008; Beaty, 2015; Boccia et al., 2015; Kasdan et al., 2022). The NMR encompasses various brain regions that process temporal, cognitive, motor, and sensory information. Regarding temporal sequences, the putamen and globus pallidus (also constituents of the basal ganglia network (BGN)) act as internal pacemakers to stabilize the beat (Nozaradan et al., 2017; Kasdan et al., 2022). The inferior frontal gyrus is responsible for the sequencing and organization of rhythms (Fadiga et al., 2009; Uddén and Bahlmann, 2012; Fitch and Martins, 2014), while the inferior parietal lobule is associated with temporal processing and handles medium complexity rhythmic patterns (Zhan et al., 2015; Kasdan et al., 2022). The supplementary motor area and pre-supplementary motor area (also constituents of the sensorimotor network (SMN)) are responsible for complex rhythmic patterns (Akkal et al., 2007;Chen et al., 2008; Kasdan et al., 2022). Regarding cognitive strategies, the superior frontal gyrus and prefrontal cortex are involved in understanding rhythmic information and strategic processing (Alluri et al., 2012; Musacchia et al., 2014). The temporal areas process auditory rhythm signals (Chen et al., 2008; Kasdan et al., 2022). Motor-related regions are linked to the execution and planning of movement sequences (Chen et al., 2008; Cannon and Patel, 2021). The superior parietal lobule serves as a sensorimotor integration zone related to attention (Kasdan et al., 2022; Sulpizio et al., 2023), while the cerebellum (part of the cerebellar network (CN)) is associated with motor coordination and timing (Nozaradan et al., 2017; Kasdan et al., 2022). Overall, the NMR plays a pivotal role in executing and generating novel motor sequences through top-down control, giving rise to creative movement patterns (Chen et al., 2008; Alluri et al., 2012; Bailey et al., 2014; Kasdan et al., 2022). The convergence of multiple neural networks within the NMR underscores that rhythm timing and production extend beyond a mere musical element to encompass a sensory experience (Chen et al., 2008; Bailey et al., 2014; Loui, 2018).

Furthermore, the default mode network (DMN) and the executive control network (ECN) are identified as key components of improvisation, mirroring the operations of internal and external cognitive process, respectively. These networks are engaged in a dynamic interplay during improvisation (Limb and Braun, 2008; Beaty, 2015; Vergara et al., 2021). It has been suggested that an increase in the DMN activity coupled with a decrease in the ECN activity suggests that performers become deeply engrossed in the musical context, plausibly entering a flow state that diminishes outward focus (Limb and Braun, 2008; Beaty, 2015). In addition to these functions, the multiple demand network and the salience network (SN) also play a key role in supporting wide range of cognitive capacities including problem solving, attention, memory processes, focusing and sensing emotional cues in the music (Beaty, 2015; Lu et al., 2017; Vergara et al., 2021). Importantly, the activation of the limbic system (LS) and its related memory systems highlights the process of recalling and connecting to past experiences, as well as the utilization of learned musical structures and emotional processing (Limb and Braun, 2008; McPherson et al., 2014). The LS houses the reward system, intricately linked to learning motivation and satisfaction (Zatorre, 2015; Gold et al., 2019). Furthermore, the language network (LAN) is involved in processing the syntax and semantics of music (Koelsch, 2005; Koelsch, 2012). These networks (i.e., NMR, DMN, AN, SMN, and other related ones) demonstrate intricate interactions across different improvisational scenarios.

Terms like “free improvisation,” “jazz,” or “blues” often describe different improvisational styles (Bailey, 1992; Ng, 2011). In percussion scores, “free improvisation” can refer to following specific patterns or a particular musical style. Percussionists’ ability to improvise rhythmically is a signature skill developed through extensive training and practice. During performances, they may employ various strategic approaches to improvisation. The strategy of structural improvisation (SIMP), as discussed in this paper, involves improvising within a specified structure or set of parameters, which can include designated rhythmic patterns. In contrast, the strategy of free improvisation (FIMP) involves improvising without relying on specific rhythmic patterns but instead adhering to the overall musical style (Bailey, 1992).

Our current study designs two experimental setups that mirror improvisation processing, focusing on both modes of improvisation strategies. SIMP is more idea-constrained, while FIMP allows for more spontaneous musical ideas. The goal is to investigate how percussionists’ brains represent SIMP and FIMP, pinpointing the commonalities and differences. These scenarios aim to shed light on how the NMR and other related networks are engaged during various improvisational contexts, deepening the understanding of how the brain handles rhythm creation and improvisation.

## 2 Materials and methods

### 2.1 Participants

Percussionists (PERCs) (university students majoring in percussion or active graduates performing in percussion ensemble) were recruited. All should have at least 12 years of percussion experience, capable of free improvisation, and actively participating in regular concert performances. They should be without any history of neuropsychiatric disorders. The study received approval from the local ethics committee of Taipei Veterans General Hospital, and all participants provided written informed consent.

### 2.2 Percussion training survey, self-report and self-assessment of improvisational performance

The participants specialized in percussion as their primary instrument. In Taiwan’s specialized art education program, students typically begin musical training in elementary school, selecting one instrument as their primary focus and exploring additional instruments as they advance through to university. To comprehensively understand participants’ percussion learning experiences and music education backgrounds, the survey gathers information on their primary instrument major, instruments learned, total years of music education, years of percussion study, age when music and percussion training began, weekly practice hours, and daily practice hours. Additionally, PERCs filled out a self-report detailing the mental processes they employed during two improvisation strategies. They also provided a self-assessment score of their satisfaction with their improvisational performance using a 10-cm visual analog scale, where 0 indicates ‘completely dissatisfied’ and 10 signifies ‘completely satisfied.’

### 2.3 Stimuli

In this study, both visual and auditory cues were employed in our experimental designs. The visual cues included six rhythmic patterns and a central fixation crosshair. These rhythmic patterns were designed in a 6/4 time signature, maintaining a consistent tempo of 100 beats per minute (BPM), where each beat corresponded to a quarter note, leading to six beats within each measure (refer to Supplementary Figure S1). The rhythmic patterns were created using Finale software (MakeMusic, Inc., Louisville, CO, United States). The visual cues were showcased on a screen during the functional magnetic resonance imaging (fMRI) scan. Participants received five measures at one time. The first measure was the rhythmic patterns and the other four measures were space for each improvisational task. On the auditory side, rhythmic patterns were set at 100 BPM, rendered at an appropriate volume via the same Finale software (MakeMusic, Inc., Louisville, CO, United States), and maintained a sampling rate of 44.1 kHz. Participants listened to the metronome sound throughout the visual cues. The volume was adjusted individually for each participant to ensure optimal auditory comfort. Participants were directed to rhythmic tapping and improvised responses using a bimanual fiber optic response pad (Current Designs Inc.) that was compatible with the MRI environment, while simultaneously interacting with the visual stimuli and listening to the auditory beat. The experiment was administered, and the reaction response registered using Presentation software version 0.71 by Neurobehavioral Systems, Inc.

### 2.4 Experimental design of fMRI experiments

**Figure 1:**
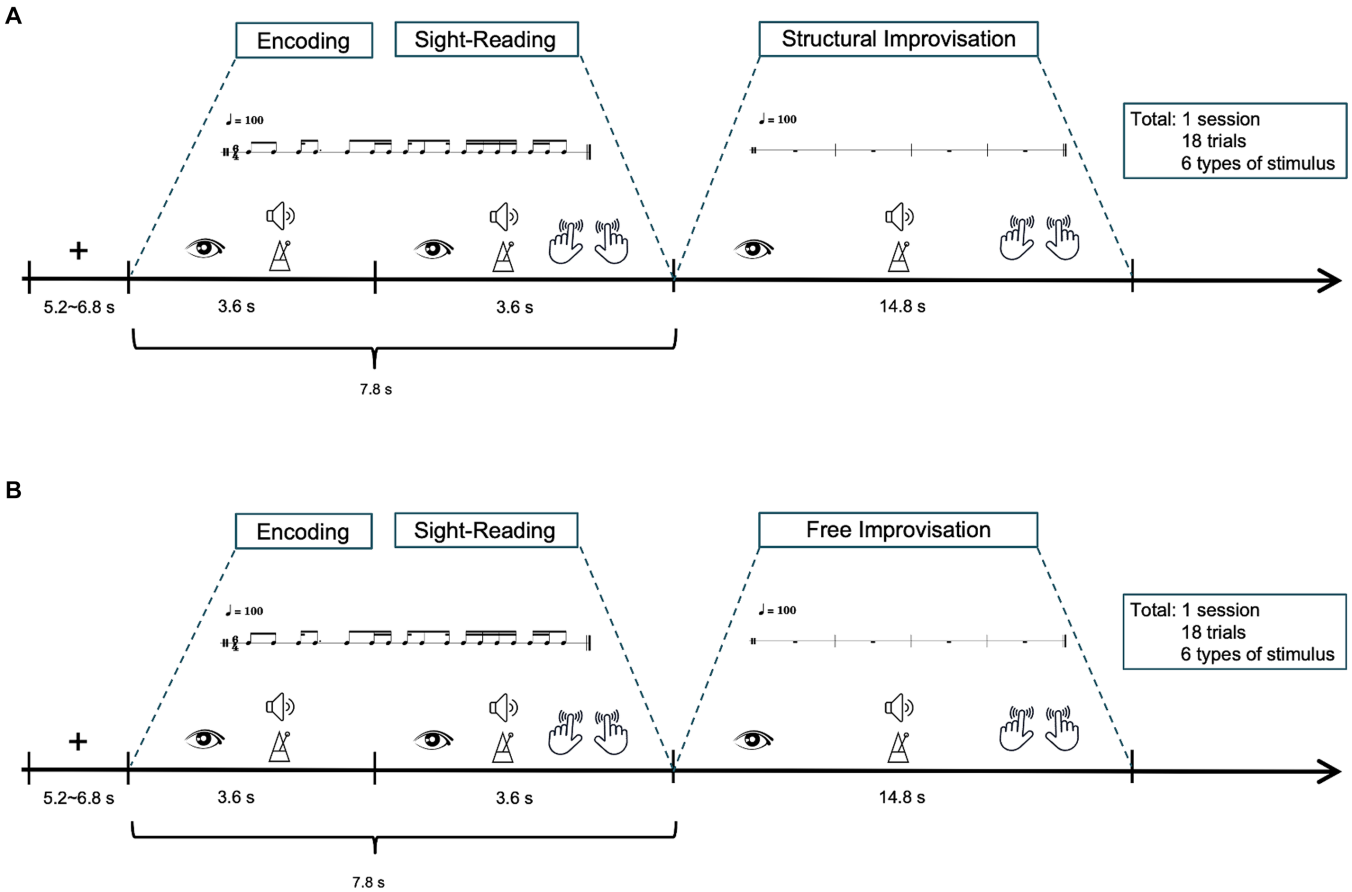
Schematic diagram of the fMRI experiment. The study involved two tasks: **(A)** structural improvisation and **(B)** free improvisation. Participants initiated the process by focusing on a white crosshair for 5.2 to 6.8 s. After that, participants view a percussion score in a 6/4 meter, accompanied by a sound beat, while maintaining a tempo of 100 BPM, referred to as the encoding (lasting 3.6 s). Following this, participants observe rhythmic notations in a 6/4 meter for 3.6 s and instructed to tap the rhythm with 100 BPM tempo, constituting the sight-reading (lasting 3.6 s). Subsequently, participants were instructed to improvise rhythms at a tempo of 100 BPM, with each improvisation session lasting 14.8 s. Each trial extends for approximately 29.4 s, and a complete session encompasses 18 trials, resulting a total duration of around 529.2 s, equivalent to 8.82 min.

Figures 1A,B illustrates the experimental setup. The fMRI study consisted of two scanning sessions, each encompassing 18 trials. Before the scanning session, participants received task instructions and had the opportunity to practice tapping the rhythm by alternating their index fingers on a touchpad. The purpose was to ensure that the participants were familiar with the tasks performed within the MRI scanner. Participants began by focusing on a white crosshair fixation point for a period of 5.2 to 6.8 s, indicating a *fixation* period. After 5.2 to 6.8 s, participants viewed percussion score in a 6/4 meter, accompanied by the sound beat, maintaining a tempo of 100 BPM throughout the 3.6-s trial, referred to as the *encoding*. Following this, participants were directed to tap the rhythm on a touchpad using their index fingers, maintaining a tempo of 100 BPM as labeled *sight-reading* (SR), and lasted 3.6 s. Following the SR task, participants proceeded to two modes of improvisation tactics: SIMP and FIMP. SIMP required participants to improvise based on the rhythmic patterns of six given examples, while FIMP allowed them to express rhythmic ideas freely, without being restricted by specific patterns. Each improvisation lasted 14.8 s. Each session consisted of 18 trials, lasting approximately 518.4 s in total, with each trial lasting about 28.8 s. The combined duration of the two sessions (SIMP and FIMP) was approximately 1036.8 s, or 17.28 min.

### 2.5 MRI data acquisition

Participants were scanned using a 3.0 T MRI machine (MAGNETOM Trio™, Siemens, Erlangen, Germany) situated at National Yang Ming Chiao Tung University, utilizing the head-coil gradient configuration. Functional imaging was conducted using T2*-weighted echoplanar imaging (EPI) techniques, featuring 40 slices with a thickness of 3.4 mm each. The specific parameters included a repetition time (TR) of 2,000 ms, an echo time (TE) of 30 ms, a field of view (FOV) measuring 220 mm × 220 mm, a voxel size of 3.4 mm × 3.4 mm × 3.4 mm, a matrix size of 64 × 64, a repetition number of 254, and a flip angle of 90^o^. Following the functional MRI segment, participants underwent a detailed T1-weighted structural scan, which had a resolution of 1 mm × 1 mm × 1 mm. This involved utilizing a magnetization-prepared rapid acquisition gradient echo (MPRAGE) method with settings including a TR of 2530 ms, TE of 3.03 ms, 1 mm slice thickness, an FOV of 224 mm × 256 mm, a flip angle of 7^o^, and a matrix size of 224 × 256, comprising 192 slices.

### 2.6 Image preprocessing

The collected imaging data were processed using the Statistical Parameter Mapping software (SPM12, Wellcome Trust Centre for Neuroimaging, University College London). MATLAB 2022a (The MathWorks, Inc., Natick, MA, United States) served as the platform for this processing. The data underwent standard preprocessing steps, encompassing slice timing adjustments, correction for head movement, alignment with the T1-weighted structural image, and spatial normalization to the Montreal Neurological Institute (MNI) standard using a typical T1 template. Additionally, spatial smoothing was applied using an 8 mm Full-Width Half Maximum (FWHM) Gaussian filter. For the slice timing adjustment, the middle slice of each scan was utilized as a reference, and any scan displaying movements beyond 3 mm translation or 3° rotation was omitted. T1 images were resampled throughout the normalization phase to have isotropic voxels measuring 2 mm in size.

### 2.7 Statistical analyses

#### 2.7.1 Self-assessment score of improvisation performance

The difference in satisfaction levels between the SIMP and FIMP was analyzed using the Wilcoxon Signed-Rank Test (SPSS Statistics Version 29, SPSS Inc., United States), where statistical significance was established at a threshold of *p* < 0.05.

#### 2.7.2 fMRI image statistics

In the 1st level (individual) analyses, regressors were structured around three conditions: fixation, SR_SIMP_/SR_FIMP_, and SIMP/FIMP, for constructing the GLM model in SPM12, based on the experimental setup. The temporal parameters for each condition were as follows: the fixation began at 0 s and persisted for about 5.2 to 6.8 s; SR_SIMP_/SR_FIMP_ started roughly at 10 s and spanned about 3.6 s; whereas SIMP/FIMP commenced between 13 to 14 s, lasting around 14.8 s. Multiple regressors based on head motion parameters from rigid-body realignment (Van Dijk et al., 2012), were included in the model. To mitigate noise, the default high-pass filter (set at 128 s) in SPM was engaged. In the 2nd level (group) analyses, each condition (SR_SIMP_, SIMP, SR_FIMP_, and FIMP) and between-condition contrasts (SR_FIMP_ vs. SR_SIMP_, SIMP vs. SR_SIMP_, FIMP vs. SR_FIMP_, and FIMP vs. SIMP) were created, respectively. One-sample *t*-test and paired *t*-test were used to evaluate all conditions. Regression procedures were applied to account for confounding variables including age and gender. All the 1st level and 2nd level analyses were confined to gray matter regions, considering results significant if the uncorrected voxel level was *p* < 0.001 and the cluster level met a family-wise error (FWE) correction of *p* < 0.05. The automated anatomical labeling (AAL) atlas was employed to pinpoint significant activation regions (Rolls et al., 2020).

## 3 Results

### 3.1 Demographic information and results of self-assessment scores

Initially, 43 PERCs were recruited for the study. After excluding individuals with incidental brain findings (anomalies or abnormalities) or significant head motion during the fMRI experiment, a final group of 25 PERCs was retained for image statistical analyses. This group had a mean age of 23.92 ± 2.84 years and included 10 males and 15 females. According to the Edinburgh Handedness Inventory (Oldfield, 1971), 23 of the participants (92%) were right-handed, while 2 were left-handed. On average, the PERCs had 18.04 ± 3.27 years of total musical training, with 15.32 ± 2.81 of those years focused on percussion. They practiced an average of 18.16 ± 9.54 h per week, dedicating approximately 2.7 ± 1.31 h each day to their training.

Self-assessment scores for the PERCs’ satisfaction with their improvisational performances after the fMRI experiment averaged 6.42 ± 1.56 for structural improvisation (SIMP) and 7.66 ± 1.15 for free improvisation (FIMP), revealing a significant difference between the two (*p* < 0.001) (Table 1).

**Table 1:** Demographic characteristics and self-rating scales.

	Percussionists	*p*-value
	(*n* = 25)	
Sex (male/female)	(10, 15)	–
Age (years)	23.92 ± 2.84	–
Education (years)	16.82 ± 1.49	–
Duration of music training (years)	18.04 ± 3.27	–
Duration of percussion training (years)	15.32 ± 2.81	–
Weekly practice (hours)	18.16 ± 9.54	–
Daily practice (hours)	2.7 ± 1.31	–
Self-rating scales		
SIMP	6.42 ± 1.56	<0.001
FIMP	7.66 ± 1.15	

Data are expressed as mean ± standard deviation. The self-rating scales for SIMP and FIMP were compared using the Wilcoxon Signed-Rank Test, with a significance level established at *p* < 0.05.SIMP, structural improvisation; FIMP, free improvisation.

### 3.2 Basic imaging analyses

To affirm active brain involvement across different conditions (SR_SIMP_, SIMP, SR_FIMP_, and FIMP), we applied the one-sample *t*-test. The results indicated changed neural activity in several networks, including the NMR, SMN, AN, BGN, CN, SN, ECN, LAN, and visual network (VN). In contrast, the DMN displayed a decline in activity. Additionally, enhanced activity was noted in the putamen and globus pallidus for both SIMP and FIMP. The details of the results from the one-sample *t*-tests for the four conditions can be referred to in Supplementary Tables S1–S4 and Supplementary Figure S2. To confirm the consistency of the baseline, a paired *t*-test was utilized to assess the difference between SR_FIMP_ and SR_SIMP_. The findings revealed no statistically significant disparities between SR_FIMP_ and SR_SIMP_ (Supplementary Figures S2A,C).

### 3.3 Comparisons between improvisation and sight-reading conditions

To examine the cognitive activities of PERCs across different improvisational and sight-reading conditions (SIMP vs. SR_SIMP_ and FIMP vs. SR_FIMP_), paired *t*-tests were applied. This approach allowed us to contrast cognitive dynamics in distinct tactics of improvisation (SIMP, FIMP), using sight-reading scenarios (SR_SIMP_, SR_FIMP_) as controls to adjust for basic cognitive components inherent to tasks other than improvisation itself.

#### 3.3.1 SIMP vs. SR_SIMP_

Enhanced activity was noted in several neural networks, including the NMR, ECN, and LAN. The NMR encompassed the SMN, AN, and CN. For the SMN, notable regions of heightened activity encompassed the right pre-supplementary motor area (BA6), right supplementary motor area (BA6), left dorsal premotor cortex (BA6), left ventral premotor cortex (BA6), along with bilateral thalamus. The AN showed enhanced activity in the bilateral superior temporal gyrus (BA22 and BA41). The CN exhibited higher activity in areas such as the “bilateral cerebellum lobule IV-V” and “left cerebellum lobule VI”, bilateral vermis lobule IV–V, and right vermis lobule VI. The ECN showed featured heightened activity in areas in the right superior frontal gyrus (BA8, the frontal eye field), right posterior part of the middle temporal gyrus (BA21), and left superior parietal lobule (BA7). The LAN exhibited higher activity in the left inferior frontal gyrus (BA44, Boca’s area).

**Figure 2:**
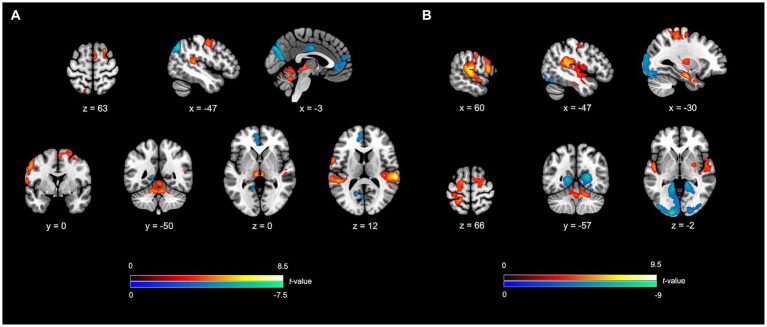
fMRI outcomes for SIMP/FIMP contrasted to SR_SIMP_/SR_FIMP_. **(A)** *SIMP vs. SR_SIMP_*; **(B)** *FIMP vs. SR_FIMP_*. In both the contrasts, the SFG (FEF), IFG, SPL, STG, SMA, dPMC, vPMC, cerebellum lobule IV-V, cerebellum lobule VI, and vermis lobule IV-V unveil higher activity. The VN demonstrate lower activity in both the contrasts. **(A)** In SIMP vs. SR_SIMP_, the pMTG, pre-SMA, Thal, and the vermis lobule VI reveal increased activity, while the DMN and SN show decreased activity. **(B)** In FIMP vs. SR_FIMP_, the mMTG, IPL (SMG), M1, S1, PUT, GP, TP, Hipp, vermis lobule VII, and vermis lobule VIII reveal the enhanced activity. Additional details regarding significant activity results and abbreviations refer to Tables 2, 3. Heightened activity is denoted by the red color, while lower activity is represented by the blue color.

In contrast, decreased activity was noted in the DMN, involving the left medial superior frontal gyrus (BA10), left anterior cingulate cortex (BA32), and left angular gyrus (BA39). The VN also demonstrated diminished activity, specifically in the left cuneus (BA19), left calcarine cortex (BA17), and left lingual gyrus (BA18). Furthermore, a reduction in activity was seen in the salience network, especially in the right middle cingulate cortex (BA23) (Table 2; Figure 2A).

**Table 2:** Outcomes comparing structural improvisation and sight-reading.

Contrast	Region	BA	Left hemisphere				Right hemisphere			
			*x*	*y*	*z*	*t-*value	*x*	*y*	*z*	*t*-value
SIMP > SR_SIMP_										
NMR										
SMN	Pre-SMA	6	–	–	–	–	4	8	60	6.13
	SMA	6	–	–	–	–	8	−2	68	4.45
	dPMC	6	−52	−4	50	7.26	–	–	–	–
	vPMC	6	−58	2	28	4.36	–	–	–	–
	Thal	–	−4	−24	0	5.98	4	−24	0	5.89
AN	STG	22	−64	−34	12	4.77	62	−30	14	8.2
	STG	41	−36	−34	12	4.03	60	−24	4	5.3
CN	Cerebellum lobule IV–V	–	−16	−50	−26	5.19	18	−44	−26	4.99
	Cerebellum lobule VI	–	−6	−66	−16	4.08	–	–	–	–
	Vermis lobule IV–V	–	−2	−60	−6	4.26	0	−50	−8	4.46
	Vermis lobule VI	–	–	–	–	–	6	−68	−18	4.54
ECN	SFG (FEF)	6/8	–	–	–	–	26	0	64	4.66
	pMTG	21	–	–	–	–	50	−48	12	4.4
	SPL	7	−16	−66	56	4.57	–	–	–	–
LAN	IFG (Boca’s area)	44	−60	6	16	4.9	–	–	–	–
SIMP < SR_SIMP_										
DMN	mSFG	10	−8	54	4	−5.05	–	–	–	–
	ACC	32	−12	42	14	−4.09	–	–	–	–
	AG	39	−46	−68	44	−7.24	–	–	–	–
VN	Cuneus	19	−4	−82	38	−7.05	–	–	–	–
	Calcarine cortex	17	−4	−70	18	−5.96	–	–	–	–
	Lingual gyrus	18	−8	−42	2	−5.38	–	–	–	–
SN	MCC	23	–	–	–	–	0	−10	36	−5.95

SIMP, structural improvisation; SR, sight-reading; BA, Brodmann’s area; NMR, network for musical rhythm; SMN, sensorimotor network; AN, auditory network; CN, cerebellar network; ECN, executive control network; LAN, language network; DMN, default mode network; VN, visual network; SN, salience network; pre-SMA, pre-supplementary motor area; SMA, supplementary motor area; dPMC, dorsal premotor cortex; vPMC, ventral premotor cortex; Thal, thalamus; STG, superior temporal gyrus; SFG, superior frontal gyrus; FEF, frontal eye field; pMTG, posterior part of the middle temporal gyrus; SPL, superior parietal lobule; IFG, inferior frontal gyrus; mSFG, medial superior frontal gyrus; ACC, anterior cingulate cortex; AG, angular gyrus; MCC, middle cingulate cortex.

#### 3.3.2 FIMP vs. SR_FIMP_

Elevated activity was observed in several neural networks, encompassing the NMR, ECN, LAN, and LS. Within the NMR, heightened activity was identified in the SMN, BGN, AN, and CN. Within the SMN, the areas of enhanced activity included the left supplementary motor area (BA6), left dorsal premotor cortex (BA6), bilateral ventral premotor cortex (BA6), right precentral gyrus (BA4), and bilateral primary somatosensory cortex (BA2/BA3). The right putamen and globus pallidus in the engaged BGN regions also form part of the reward system. The AN exhibited higher activity in the bilateral superior temporal gyrus (BA22 and BA41). The regions in the CN involved the left vermis lobule IV–V, right vermis lobule VII, left vermis lobule VIII, bilateral cerebellum lobule IV-V, and right cerebellum lobule VI. The ECN showed activations in the bilateral superior frontal gyrus (BA6 and BA8, the frontal eye field), left middle part of the middle temporal gyrus (BA21), right inferior parietal lobule (BA40, supramarginal gyrus), and left superior parietal lobule (BA7). The LAN demonstrated the higher activity in the right inferior frontal gyrus (BA44, Boca’s area). Moreover, the LS featured heightened activity in areas, including the left temporal pole (BA38) and left hippocampus (part of the memory system). Conversely, the VN demonstrated a reduction in activity, notably in the bilateral cuneus (BA18/BA19), left lingual gyrus (BA18), and bilateral calcarine cortex (BA17/BA18) (Table 3; Figure 2B).

**Table 3:** Outcomes comparing free improvisation and sight-reading.

Contrast	Region	BA	Left hemisphere				Right hemisphere			
			*x*	*y*	*z*	*t*-value	*x*	*y*	*z*	*t*-value
FIMP > SR_FIMP_										
NMR										
SMN	SMA	6	−18	−8	66	5.02	–	–	–	–
	dPMC	6	−20	−20	70	5.52	–	–	–	–
	vPMC	6	−54	2	10	5.63	60	6	32	4.57
	M1	4	–	–	–	–	32	−20	54	3.81
	S1	2/3	−22	−40	66	5.73	60	−12	46	5.02
AN	STG	22	−60	−34	12	9.16	54	−6	−6	6.18
	STG	41	−42	−36	14	6.76	54	−26	8	7.81
CN	Vermis lobule IV–V	–	−2	−56	−12	3.72	–	–	–	–
	Vermis lobule VII	–	–	–	–	–	2	−62	−26	4.88
	Vermis lobule VIII	–	−2	−64	−34	4.34	–	–	–	–
	Cerebellum lobule IV–V	–	−10	−54	−22	4.05	16	−50	−22	4.63
	Cerebellum lobule VI	–	–	–	–	–	22	−54	−26	4.79
BGN	PUT	–	–	–	–	–	32	−6	4	4.52
	GP	–	–	–	–	–	28	−4	−4	4.49
ECN	SFG (FEF)	6/8	−24	−10	62	4.23	20	−4	72	5.02
	mMTG	21	−50	−8	−22	5.28	–	–	–	–
	IPL (SMG)	40	–	–	–	–	64	−20	32	4.85
	SPL	7	−24	−46	66	4.9	–	–	–	–
LAN	IFG	44	–	–	–	–	58	8	22	5.76
LS	TP	38	−30	6	−34	5.18	–	–	–	–
MS	Hipp	–	−30	−14	−22	6.91	–	–	–	–
FIMP < SR_FIMP_										
VN	Cuneus	18/19	−2	−86	36	−8.04	10	−78	20	−7.57
	Lingual gyrus	18	−4	−68	6	−6.88	–	–	–	–
	Calcarine cortex	17/18	−18	−60	4	−7.08	2	−72	16	−6.99

FIMP, free improvisation; BGN, basal ganglia network; LS, limbic system; MS, memory system; M1, primary motor cortex; S1, primary sensorimotor cortex; PUT, putamen; GP, globus pallidus; mMTG, middle part of the middle temporal gyrus; IPL, inferior parietal lobule; SMG, supramarginal gyrus; TP, temporal pole; Hipp, hippocampus; also refer to Table 2 for other abbreviations.

#### 3.3.3 FIMP vs. SIMP

**Figure 3:**
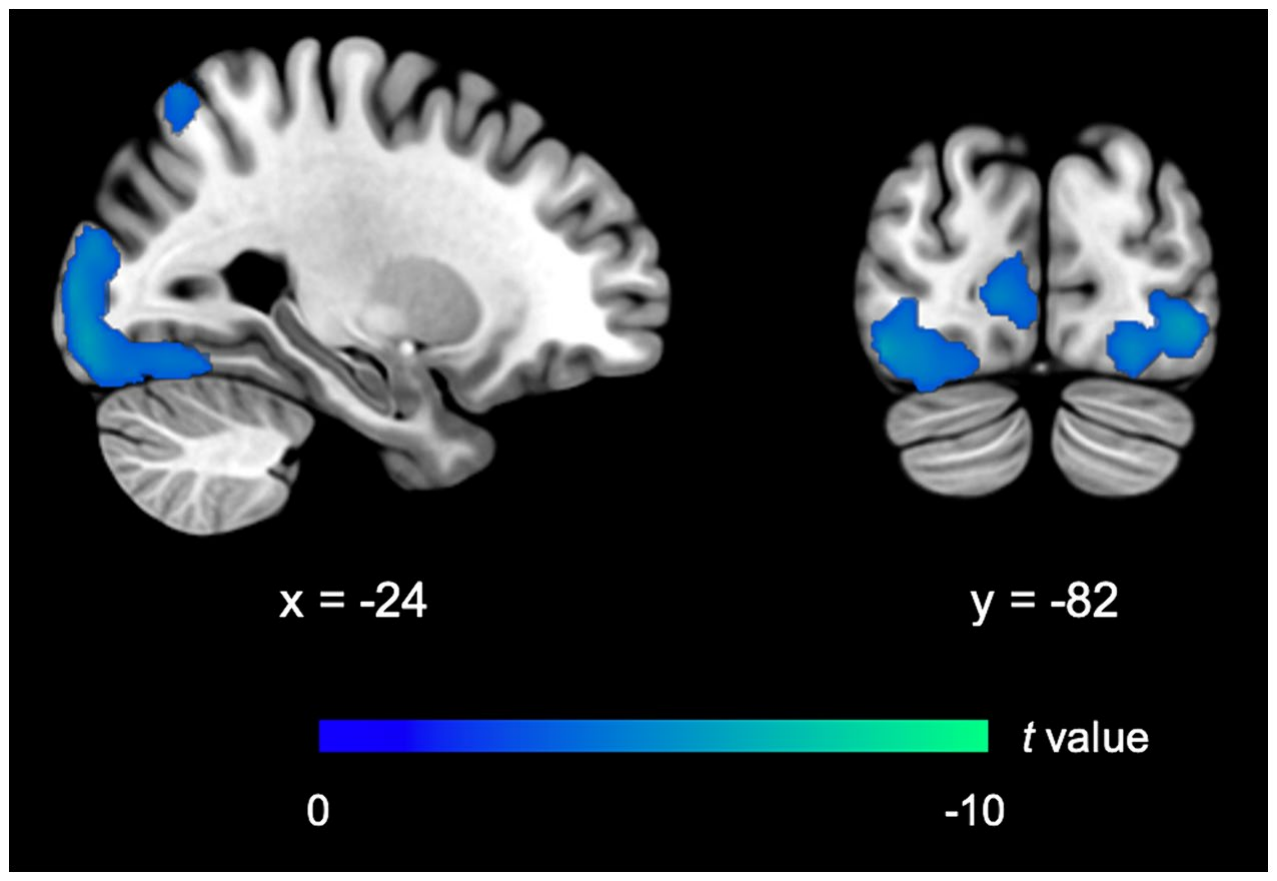
fMRI outcomes for FIMP contrasted to SIMP. The imaging findings exhibit decreased activity in the ECN and VN. Further details on significant activity and abbreviations can be found in Table 4. Areas of decreased activity are marked in blue.

FIMP, compared to SIMP, demonstrated a significant reduced activity in the ECN (i.e., left superior parietal lobule, BA7). Activity in the VN, including the bilateral calcarine cortex (BA17/BA18), bilateral inferior occipital gyrus (BA18/BA19), left middle occipital gyrus (BA18), left lingual gyrus (BA18), and left fusiform gyrus (BA19/BA37) was relatively reduced. There was no observable significant difference in the LS (including memory and reward systems), implicating that brain activity levels were comparable between FIMP and SIMP. Compared to SIMP, there was no notable rise in brain activity observed in FIMP (Table 4; Figure 3).

**Table 4:** Outcomes of comparing free improvisation and structural improvisation.

Contrast	Region	BA	Left hemisphere				Right hemisphere			
			*x*	*y*	*z*	*t*-value	*x*	*y*	*z*	*t*-value
FIMP > SIMP										
	NS	–	–	–	–	–	–	–	–	–
FIMP < SIMP										
ECN	SPL	7	−24	−68	64	−4.85	–	–	–	–
VN	Calcarine cortex	17/18	−10	−100	−4	−6.83	18	−98	−2	−9.72
	IOG	18/19	−44	−72	−18	−4.54	30	−90	−8	−9
	MOG	18	−36	−90	−2	−7.19	–	–	–	–
	Lingual gyrus	18	−38	−92	−14	−6.02	–	–	–	–
	Fusiform gyrus	19/37	−30	−64	−14	−5.63	–	–	–	–

NS, not significant; also refer to Tables 1, 2 for the abbreviations.

## 4 Discussion

**Figure 4:**
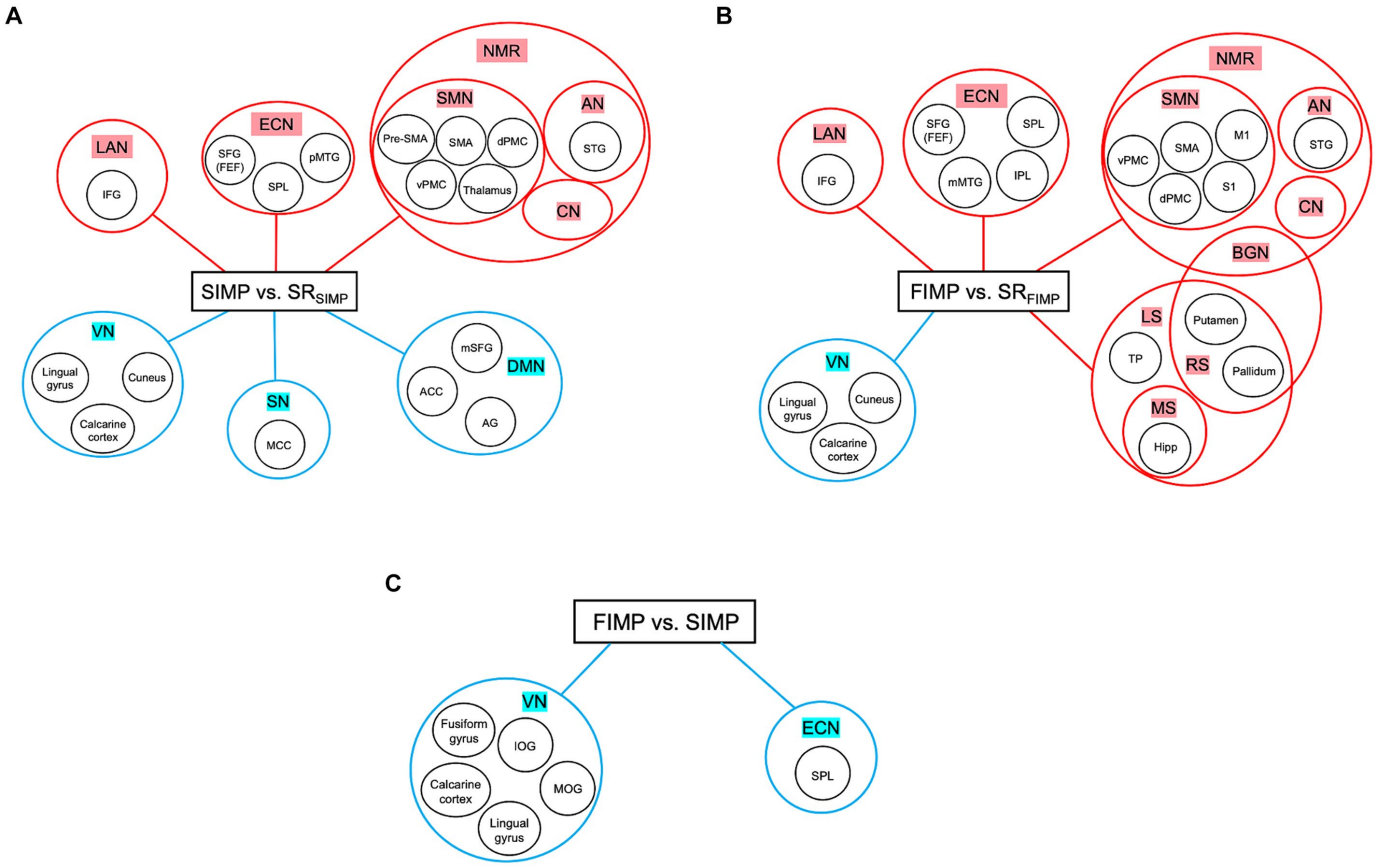
Summary of three contrasts. **(A)** SIMP vs. SR_SIMP_. **(B)** FIMP vs. SR_FIMP_. **(C)** FIMP vs. SIMP. In both SIMP vs. SR_SIMP_ and FIMP vs. SR_FIMP_, the NMR and ECN play a significant role. Moreover, the increased in LAN indicates that caveat humming techniques are unique for PERCs, while the decreased VN shows minimally engage with external visual cues. **(A)** In SIMP, increased ECN and decreased SN and DMN suggest that PERCs maintain a delicate balance between internal rhythm organization and external message constraints with creative freedom for organizing the rhythms. **(B)** In FIMP, the PUT and GP are part of BGN and RN, reflecting the inner sense of rhythm and motivational movement. Moreover, the rise in ECN, LS, MS, and RS implies that PERC draws inspiration from familiar rhythmic patterns and organizes these rhythmic concepts for improvisational creation. **(C)** In the comparison between FIMP and SIMP, the decreased SPL within the ECN and VN indicates that PERCs reduce their attentional perception of external visual input. Abbreviations refer to Tables 2–4. Heightened activity is denoted by the red color, while lower activity is represented by the blue color.

The high levels of satisfaction with their improvisation performance reported by percussionists in both SIMP and FIMP reflect their active engagement with the task (Table 1). In SIMP, the dynamic interplay among the NMR, ECN, and DMN is crucial for percussionists’ rapid creation of novel rhythmic patterns under specific set of constraints. In contrast, during FIMP, the enhanced cooperation among the NMR, ECN, LS, MS, and RS underscores the significant reliance on past experiences by percussionists to foster creative endeavors. This differentiation in neural mechanisms during SIMP and FIMP, as exhibited by percussionists through intensive practice, align with Loui’s (2018) theory at both the algorithmic and implementational levels, highlighting the varied cognitive strategies percussionists adopt to nurture creativity in improvisation (Loui, 2018) (Figures 4A–C).

### 4.1 Common neural implementations for the two modes improvisation strategies

#### 4.1.1 The NMR and ECN as foundational neural systems for improvisation mastery

Our findings reveal that the NMR, which engages the SMN, AN, and CN in the current study (Kasdan et al., 2022), plays a pivotal role in both SIMP and FIMP (Tables 2, 3). Percussionists employ rhythmic strategies to manipulate rhythm arrangements, sequences, and structures, aiming for goal-directed performance, demonstrating the sophisticated use of rhythm in their craft (Slater et al., 2017; Loui and Guetta, 2019). In the context of rhythmic improvisation, the NMR is instrumental in managing musical components, thereby facilitating creative expression within a performance (Beaty, 2015). Concurrently, the ECN is critical for the formulation and implementation of musical strategies, underscoring its role in higher-level cognitive processing (Chen et al., 2013; Shen et al., 2016). Specifically, the AN specializes in processing beat information, crucial for rhythm perception and generation (Schlaug et al., 2005; Schlaug, 2015; Kasdan et al., 2022). Meanwhile, the SMN alongside the CN is tasked with the precise control of intricate finger movements and the processing of sensorimotor timing, essential elements for the execution of improvised rhythms (Sokolov et al., 2017; Kasdan et al., 2022). The synergistic activation of the NMR and ECN in PERCs underscores a remarkable level of cognitive engagement, enabling the use of complex rhythmic strategies and the adaptable application of rhythm in improvisational contexts (Figures 4A,B). This synthesis underscores the intricate neural mechanisms underpinning rhythmic improvisation, illustrating how percussionists leverage a sophisticated network of brain regions to achieve artistic creativity and precision in performance.

#### 4.1.2 Caveat humming as a cognitive strategy in improvisation

Caveat humming, or the mental rehearsal of rhythms, emerges as a pivotal strategy for PERCs during both SIMP and FIMP, facilitated by the activation of Broca’s area (BA44) in the LAN, highlighting the role of rhythm syntax in music cognition (Koelsch, 2005; Koelsch, 2012). Furthermore, activation in the middle temporal gyrus is in line with its importance for rhythmic understanding and mental rehearsal (Schlaug et al., 2005; Schlaug, 2015). The neurological manifestation is corroborated by the self-report of PERCs that they either counted internally, articulated about the intended rhythmic patterns, or recalled rhythms they had practiced. This internal vocalization aids in the encoding and execution of musical pieces, leveraging the auditory and motor systems to enhance musical precision and timing. Caveat humming during music performance, such as on piano or drums, is where musicians internally vocalize rhythmic patterns to improve the execution of pieces, aiding in maintaining tempo and coherence (Racette and Peretz, 2007). This leverages the auditory and motor systems, enhancing music cognition and language processing (Bangert et al., 2006; Sluming et al., 2007), thereby increasing precision and expressiveness in bridging musical ideas and performance practice (Keller, 2012; Bishop, 2018). This technique illustrates the neurological basis of rhythm processing, especially the activation of the Broca’s area in the LAN, emphasizing rhythm’s role in music cognition (Koelsch, 2005; Koelsch, 2012). During improvisation, this mental rehearsal helps musicians organize rhythmic patterns, essential for creativity (Barrett, 1998).

#### 4.1.3 Subordinate visual role in improvisation

Interestingly, both SIMP and FIMP showed decreased activity in the VN compared to respective SR scenario (Tables 2, 3; Figures 2, 4A,B), with FIMP exhibiting a more pronounced decline (Table 4; Figures 3, 4C). This suggests a minimal reliance on visual cues as also evidenced by the self-report of PERCs, pointing toward a more auditory and kinesthetic engagement, emphasizing the primacy of sound and movement in the creative exploration of rhythms.

### 4.2 Dynamics of cognitive networks in SIMP: evidence of externally directed cognition through ECN, DMN, and SN interactions

In the context of SIMP as opposed to SR_SIMP_, PERCs performed improvisation within specific rhythmic frameworks, requiring detailed coordination among the ECN, DMN, and SN—networks integral to advanced cognitive functions. During SIMP, notable changes in network activity were recorded: increased ECN activity and decreased DMN activity (Table 2; Figure 2A), indicating a pivot toward externally focused cognitive efforts in organizing rhythms and generating concepts (Liao et al., 2024). This highlights how PERCs utilize cognitive strategies to navigate the limitations imposed by SIMP, yet retain creative liberty. The SN plays a pivotal role in regulating the ECN and DMN, aiding the transition between external and internal cognitive oversight, which influences rhythmic structuring and the creation of musical ideas (Sridharan et al., 2008). A reduction in SN activity, especially in the middle cingulate cortex, reflects a nuanced equilibrium in internal rhythm management within set constraints. This complex dynamic is consistent with PERCs’ self-reports, which reveal a periodic concentration on aspects of the prescribed rhythmic framework for structuring rhythms and the adoption of improvisational tactics that shift away from the internal focus typically linked with decreased DMN activity (also illustrated in Figure 4A). Such a constraint could lead to reduced satisfaction levels in improvisation outcomes during SIMP relative to FIMP, as evidenced by the self-rating scores.

### 4.3 Dynamics of cognitive network in FIMP

The FIMP represents a form of spontaneous artistic expression, distinguished by its lack of pre-determined structures or content. Instead, it weaves together past musical experiences and current emotional states, creating an improvised musical narrative (Barrett, 1998; Biasutti and Frezza, 2009). This creative process heavily relies on rhythm and tempo to structure the music, ensuring coherence and harmony in the performance (Wang, 1984; Barrett, 1998). In FIMP, percussionists often draw on familiar rhythmic patterns, introducing variations to express creativity.

#### 4.3.1 Rhythmic stability and internal motivation in FIMP

The BGN, particularly the putamen and globus pallidus, is crucial for rhythmic processing and maintaining tempo stability (Grahn, 2009; Kasdan et al., 2022). Enhanced activity in these areas reflects the percussionists’ ability to keep a stable internal beat, which is vital for the improvisational coherence (Liao et al., 2024). Additionally, these structures are also implicated in motoric motivation (Schultz, 2016) and exploring rhythms, supporting the idea that FIMP involves navigating past experiences and present feelings to create music.

#### 4.3.2 Synthesis of sensory inputs, past experience with current creative expression

The simultaneous activation of the temporal pole and the hippocampus during FIMP reveals a sophisticated process that blends sensory perception, emotional depth, and memory retrieval (Voss et al., 2017; Mesulam, 2023). This interplay may transform percussion into a pleasant experience, showcasing improvisation as an endeavor that extends beyond mere technical proficiency to musical spontaneity. It may combine remembered rhythms and personal sentiments, illustrating the temporal pole’s key role in amalgamating cognitive and emotional components for emotionally resonant and technically sound performances. The self-reports of the PERCs and the simultaneous engagement of the hippocampus align with studies emphasizing the hippocampus’s role in drawing from past musical experiences to foster innovation and anticipation in improvisation. This implicates an exploration of both familiar and novel rhythmic domains (Voss et al., 2017; Bastiaansen et al., 2019; Zhang et al., 2020).

The ability of percussionists to freely select and invent rhythms, guided by their immediate emotional and creative impulses, demonstrates the crucial role of practice in sharpening improvisational talent. Participants’ tendencies to draw on and creatively modify familiar rhythmic patterns, as per their self-reports, further emphasize the fundamental connection between disciplined practice and the enhancement of creative musical output. This synthesis of practice, emotional resonance, and creative liberty in FIMP underlines the indispensable value of experiential learning in the mastery of improvisational skills. Regular practice significantly contributes to the spontaneity and adaptability in musical expression (Chen et al., 2008; Giovannelli et al., 2014).

#### 4.3.3 Absence of DMN variation in FIMP

Our study found no significant difference in DMN activity between FIMP and SR_FIMP_ despite observing a notable decrease in DMN activity during a single FIMP condition (Supplementary Table S4; Supplementary Figure S2D), contrasting with the conditions found in SIMP and SR_SIMP_. The discrepancy is attributed to the inherently more introspective cognitive spontaneity characterizing FIMP, as evidenced by PERCs’ self-reports indicating greater spontaneity in musical ideation and deeper engagement in an automatic flow, even within the brief duration of improvisation (approximately 14.8 s). This condition is linked to increased (or less diminished) activity in the DMN (Limb and Braun, 2008; Chialvo et al., 2014; Rosen et al., 2020).

### 4.4 Direct comparisons between FIMP and SIMP

In comparison to SIMP, FIMP was characterized by an additional decrease in ECN and VN activity (Table 4). This supports PERCs’ self-reports of frequently closing their eyes to fully engage with the improvisational music flow. The decrease in ECN activity may be attributed to a lower level of externally directed focus in FIMP compared to SIMP. The absence of significant DMN fluctuations suggests that the distinction in DMN activity between FIMP and SIMP could be minimal, possibly due to the experiment’s design involving brief improvisation sessions that limit PERCs’ ability to experience deep flow states.

## 5 Further consideration and limitations

We recognize that musical aptitude tests evaluate an individual’s potential in music. We did not conduct these tests for several reasons. Firstly, studies examining the relationship between musical aptitude and improvisation performance have yielded controversial results. Some research has found that musical aptitude scores can predict improvisation skills (Benedek et al., 2014), while other studies have found no significant correlation (McPherson and Gabrielsson, 2002) or even negative associations (Grabner et al., 2017). It is argued that musical aptitude may reflect conventional musical knowledge and skills, which could hinder the generation of novel and original musical ideas. Secondly, the PERCs enrolled in the current study had developed professional rhythmic proficiency through dedicated training under a specialized art education system in Taiwan for more than 12 years since early childhood. In addition to the inclusion criteria, their self-reports and the performance satisfaction scores after the fMRI experiment indicated that they followed the instructions faithfully. However, our current neuroimaging study could not resolve the intricacies between musical aptitude and musical achievement. Musical aptitude refers to an individual’s inherent potential in music, while music achievement pertains to the skills and knowledge gained through dedicated practice and effective education. Future research is needed to understand how musical aptitude shapes SIMP and FIMP in PERCs.

Improvising in the noisy MRI scanner imposed unprecedented challenges on the participants. In the fMRI experiment, participants were asked to tap on a finger pad with alternating taps using both index fingers while lying down in a very constricted tunnel, which differs significantly from using conventional drumsticks or both hands on an African drum. Consequently, they could not use techniques like rolls, accents, or flams. Under these constrained conditions, participants conveyed their improvised rhythmic patterns solely through fingertip movements, making it impossible to achieve the same effect as in actual performances. Furthermore, the transcription method in this study recorded the time intervals of each rhythmic pattern using the finger pad (measured in milliseconds) rather than real-time audio recordings. As a result, the rhythmic patterns transcribed from the time points may have some discrepancies compared to their real-time production. Furthermore, the PERCs in this study primarily engage in improvisation during ensemble sessions or with percussion compositions, contrasting with jazz improvisers who prioritize a more free and spontaneous expression of musical ideas. This divergence in educational backgrounds likely influences the adoption of unique improvisational strategies (Biasutti, 2015, 2017), which could explain the differences observed compared to previous research findings (Limb and Braun, 2008; Chialvo et al., 2014; Beaty, 2015).

**Figure 5:**
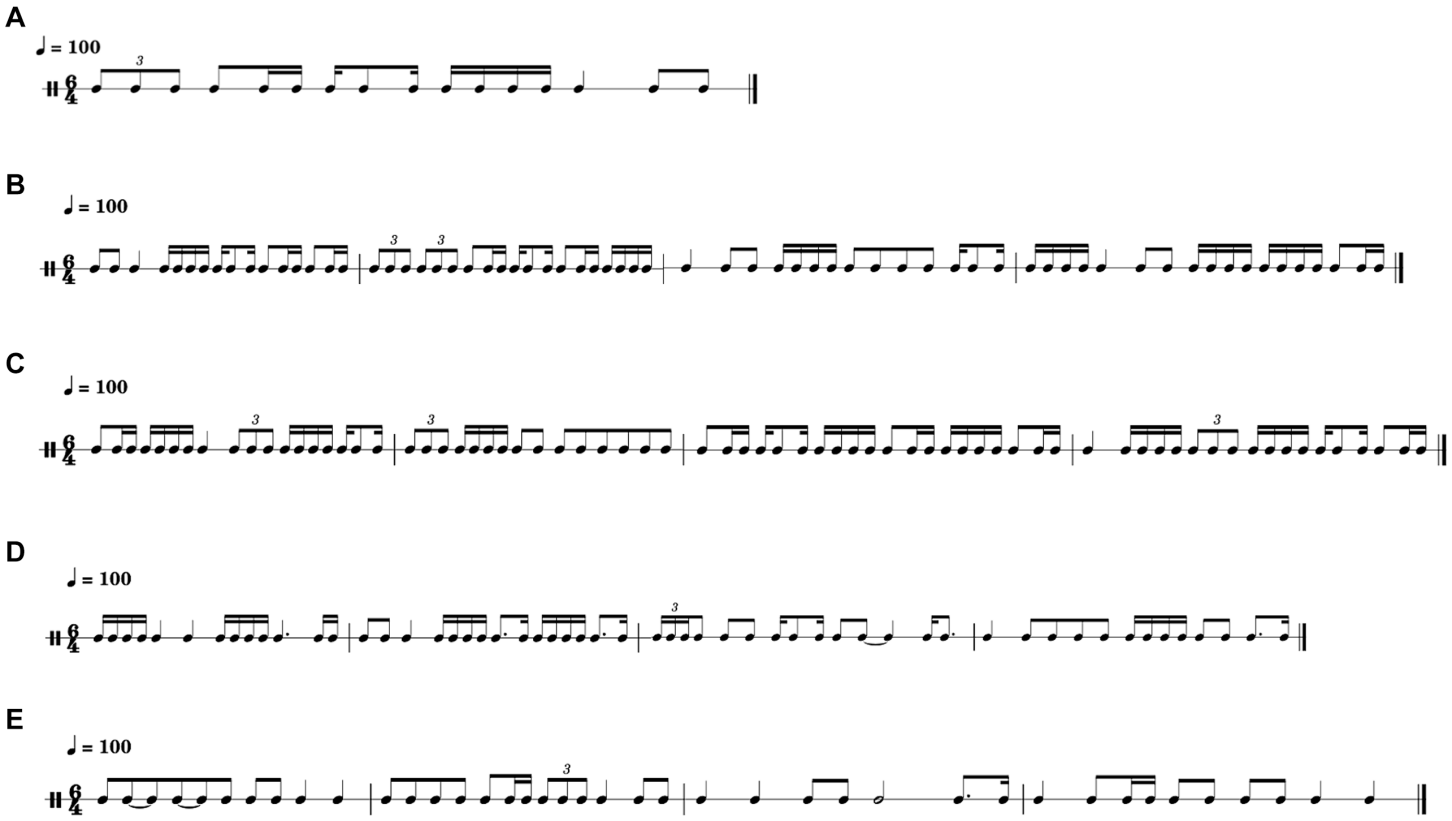
Examples of rhythmic improvisation during the SIMP and FIMP. **(A)** Illustrates one of the stimulus materials utilized in the SIMP. **(B,C)** Showcase improvisations by participant No. 22 and 24 during the SIMP. **(D,E)** Feature improvisations by participant No. 24 and 30 during the FIMP. Each measure follows a 6/4 m with a tempo set at 100 BPM. Participants improvised four measures, lasting for 14.8 s. The rhythmic notations are created using music notation software (MuseScore 4.1.1, MuseScore). SIMP, structural improvisation; FIMP, free improvisation; BPM, beats per minute.

We did not explicitly score the improvisation performance using expert evaluators, nor was our study designed to assess variations in improvisation quality between subjects. Instead, our focus was on probing the subtle brain processing differences when percussionists adopted different improvisation strategies, specifically SIMP versus FIMP. SIMP is more idea-constrained, while FIMP allows for more musical spontaneity. This distinction was reflected in self-rated performance satisfaction, as revealed by our data. Figure 5 illustrates that the participants did not merely repeat parts of the rhythms. Generally, SIMP retained more motifs and elements from the given stimulus, while FIMP showcased more spontaneous musical ideas. The participants also reported feeling significantly happier with the free improvisation compared to the structural one, as indicated by their self-rating scores of satisfactions. However, this satisfaction does not imply that the rhythms they produced were more aesthetically pleasing and creative, which would require a different evaluation. The focus of this study was not on the aesthetic quality of the rhythms.

Since the aesthetic quality of the improvisation is not the main focus of this study; therefore, we cannot address which strategy yields more creative outcomes. Moreover, the touch pad recordings did not capture other aesthetic features (such as timbre, dynamic soft-loud range, beats, etc.) that make the improvisation more expressive. In real situations, improvisers use both SIMP and FIMP strategies in a flexible, dynamic, conscious, and unconscious way. It is very hard to isolate which strategies lead to more pleasing and creative outcomes in the whole improvisation process.

Additionally, due to the time constraints of our experimental setup, which were necessary to obtain sufficient trials for statistical power, we could not explore the “psychological flow state” in either SIMP or FIMP status. Investigating how this state influences creative expression and cognitive functioning might require extended improvisational sessions or detailed tracking of the emotional and cognitive states of percussionists, as well as assessing the aesthetic quality of their improvisation outputs. Future studies could aim to lengthen the experiment’s timeframe, allowing an opportunity to achieve a flow state during the improvisational exercises.

## 6 Conclusion

Our investigation sheds light on the complex strategies percussionists applies across diverse improvisational forms. In rhythmic improvisation, there’s a notable interplay among the NMR, ECN, and various brain networks. Specifically, in SIMP, the dynamic interplay among the NMR, ECN, and DMN is crucial for percussionists’ rapid creation of novel rhythmic patterns under specific set of constraints. On the other hand, FIMP reveals an upsurge in the NMR, ECN, and LS (including memory and reward systems) activity, emphasizing the crucial roles of motivation and memory in swiftly generating novel rhythmic patterns within established parameters. The diminished VN activity during FIMP, as opposed to SIMP, illustrates a lesser reliance on external visual stimuli in FIMP, despite both tactics incorporating visual rhythmic elements. This study suggests the potential for various improvisation tactics to engage distinct neural pathways.

## Data availability statement

The original contributions presented in the study are included in the article/Supplementary material, further inquiries can be directed to the corresponding author.

## Ethics statement

The studies involving humans were approved by Taipei Veterans General Hospital. The studies were conducted in accordance with the local legislation and institutional requirements. The participants provided their written informed consent to participate in this study.

## Author contributions

Y-CL: Conceptualization, Methodology, Writing – original draft, Writing – review & editing, Formal analysis, Investigation, Validation, Visualization. C-JY: Conceptualization, Investigation, Methodology, Visualization, Writing – review & editing. H-YY: Resources, Writing – review & editing. C-JH: Methodology, Writing – review & editing. T-YH: Investigation, Writing – review & editing. W-CL: Investigation, Writing – review & editing. L-FC: Conceptualization, Funding acquisition, Methodology, Writing – review & editing. J-CH: Conceptualization, Funding acquisition, Methodology, Project administration, Resources, Supervision, Writing – original draft, Writing – review & editing.
