# Supplementary material for: The rhythmic mind: brain functions of percussionists in improvisation
Source: Front Hum Neurosci. 2024 Jul 15;18:1418727. doi: 10.3389/fnhum.2024.1418727 (PMC11308212; doi:10.3389/fnhum.2024.1418727)
Supplement: Supplementary file 1 [file Data_Sheet_1.pdf]

Supplementary Material

The Rhythmic Mind: Brain Functions of Percussionists in Improvisation

Yin-Chun Liao, Ching-Ju Yang, Hsin-Yen Yu, Chiu-Jung Huang, Tzu-Yi Hong, Wei-Chi Li, Li-Fen Chen[1], Jen-Chuen Hsieh *

*** Correspondence:** Jen-Chuen Hsieh, MD, Ph.D.: jchsiehibru@nycu.edu.tw

1 Supplementary Figures and Tables

1.1 Supplementary Figures

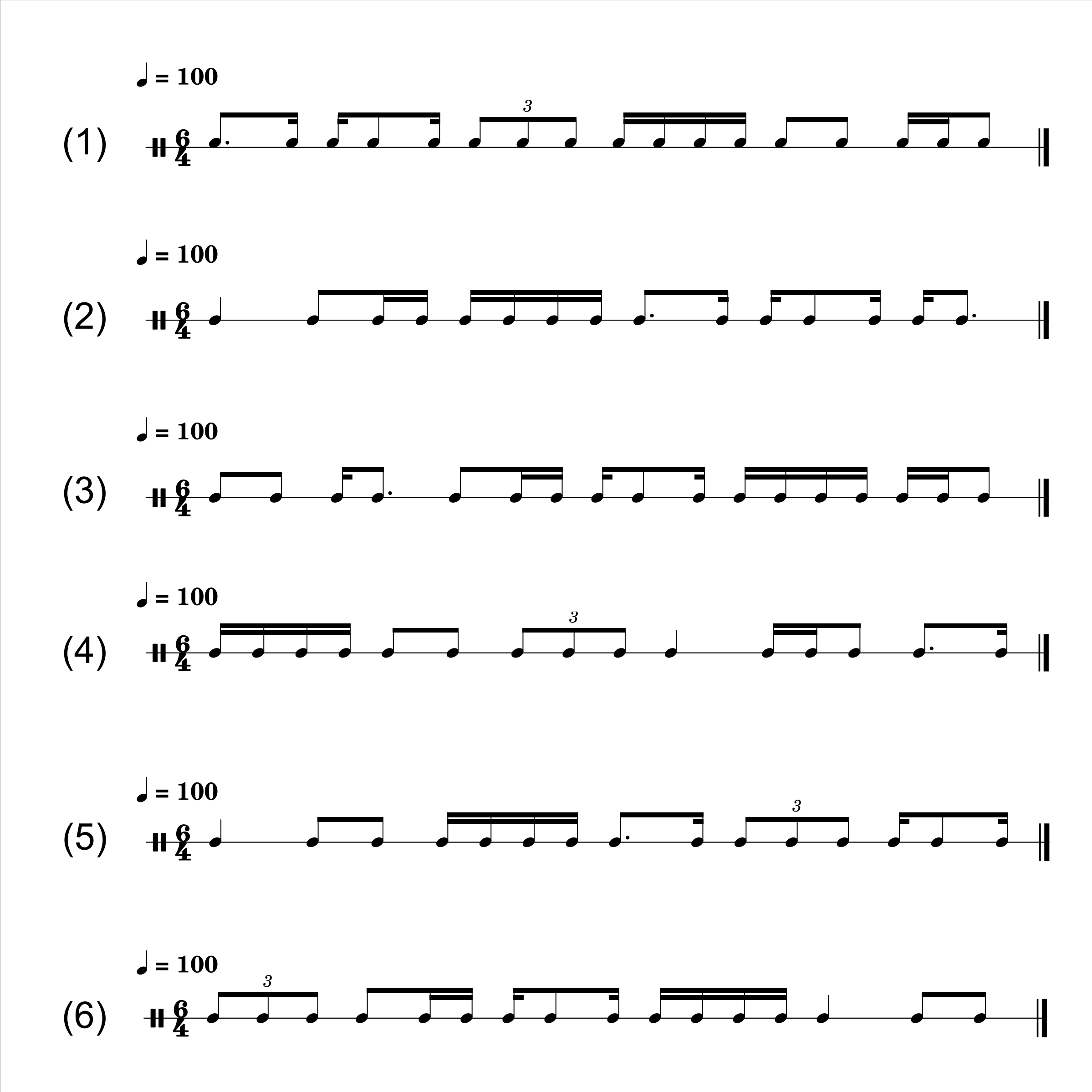

Supplementary Figure 1. Visual stimuli of rhythmic patterns. The six stimuli are set in a 6/4 meter at a tempo of 100 BPM, which equates to six beats per measure, where each beat corresponds to a quarter note. These stimuli include quarter, eighth, sixteenth, and triplets. These rhythmic patterns were generated using the MuseScore 4.1.1 software (MuseScore 4.1.1, MuseScore).

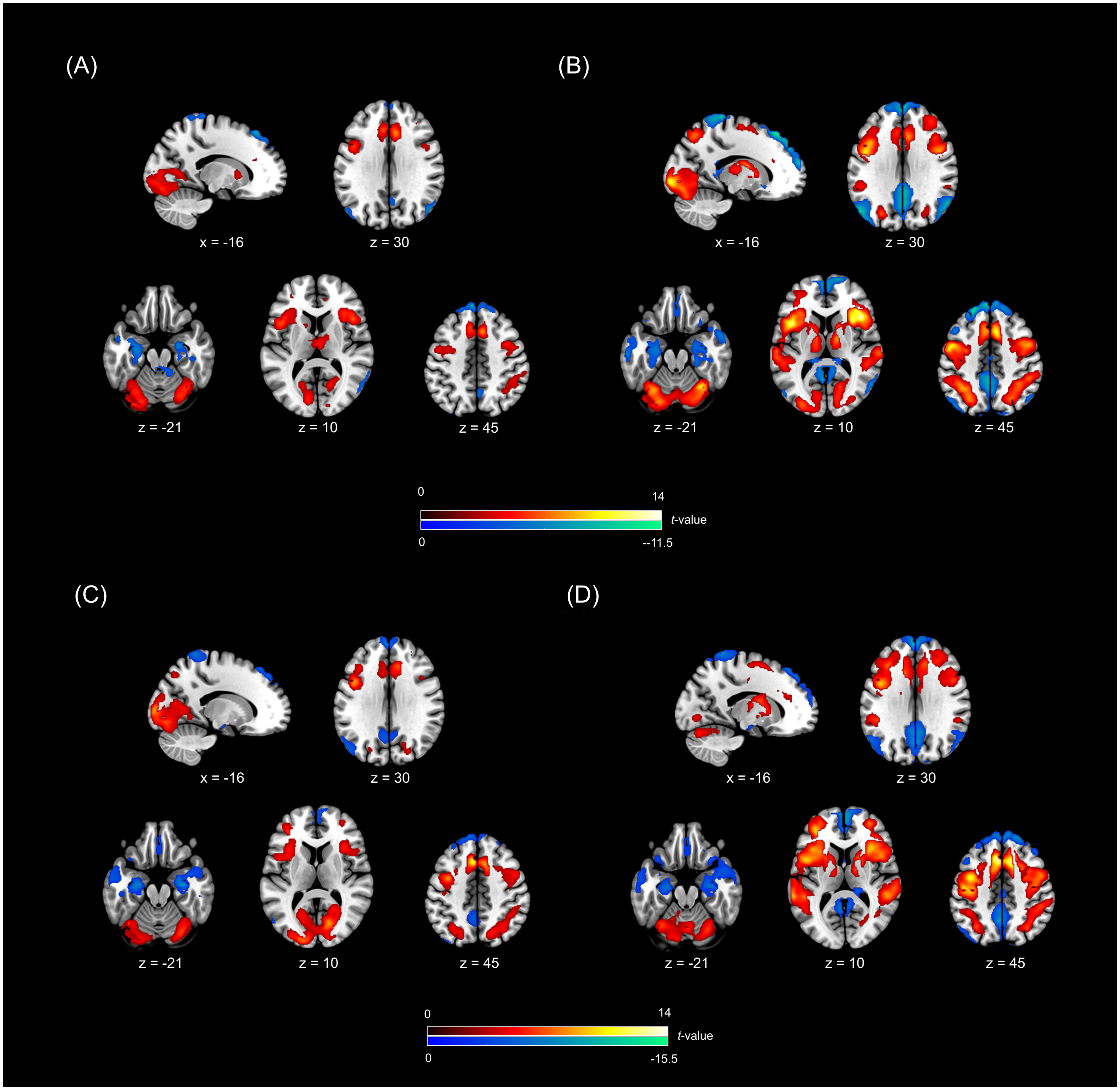

Supplementary Figure 2. Brain activations and deactivations in SR_{SIMP}, SIMP, SR_{FIMP}, and FIMP. (A) ***SR_{SIMP}*; (B) *SIMP*; (C) *SR_{FIMP}*; *(D) FIMP*.** In all four conditions, the NMR, SMN, BGN, CN, ECN, SN, LAN, and VN display increased activity, while the DMN shows decreased activity. The CN exhibits increased activity in the SR_{FIMP}, SIMP, and FIMP scenarios. In SR_{SIMP}, the region in LS is the bilateral temporal pole. The cerebellum lobule VI within the CN shows increased activity, while vermis lobule I-II, cerebellum lobule IV-V, and vermis lobule III regions within the CN demonstrate decreased activity in SR_{SIMP}. Additionally, the AN demonstrates higher activity in SIMP and FIMP. The bilateral orbitofrontal cortex, which is part of RS, shows lower activity in SIMP and SR_{FIMP}. In FIMP, the PUT and GP, which are part of RS, displays enhanced activity.

The red color represents higher activity, while the blue color denotes lower activity. All visual representations conform to neurological standards. Additional details regarding significant activity results and abbreviations refer to **Supplementary Table 1-4.**

1.2 Supplementary Tables

Supplementary Table 1. Peak MNI coordinates of the regions exhibiting significant results in SR_{SIMP}

Results	Region	BA	Left Hemisphere				Right Hemisphere			
			x	*y*	*z*	*t*-value	*x*	*y*	*z*	*t*-value
Activations										
NMR										
SMN	Pre-SMA	6	-8	4	64	6.1	10	16	56	5.45
	PMC	6	-44	-6	60	6.06	40	2	50	5.55
	Thal	-	-8	-4	4	5.87	6	-12	10	5.4
CN	Cerebellum lobule VI	-	-28	-68	-20	7.2	36	-62	-26	8.05
BGN	Caudate	-	-10	10	2	5.28	-	-	-	-
ECN	MFG	6	-30	-2	52	5.71	38	4	44	6.12
	MFG	9	-	-	-	-	38	38	22	5.44
	SFG	6	-24	0	50	5.22	-	-	-	-
	SFG	8	-10	22	48	6.7	6	20	44	8.34
	SFG	10	-	-	-	-	32	52	18	4.95
	IPL (AG)	39	56	-36	50	6.17	-	-	-	-
SN	Insula	13	-30	20	-4	6.9	34	20	-8	5.58
	MCC	32	-6	28	32	7.42	-	-	-	-
	MCC	24	-6	14	26	4.45	-	-	-	-
LAN	IFG	44	-48	8	6	7.53	44	16	10	7.21
	IFG	45	-40	18	10	6.43	45	26	8	6.34
	IFG	47	-	-	-	-	50	24	-8	4.96
VN	Lingual gyrus	18	-22	-82	-16	7.09	22	-84	-8	7.93
	Calcarine cortex	18	-8	-86	4	7.32	-	-	-	-
	Fusiform gyrus	37/19	-36	-80	-22	7.14	30	-78	-16	7.08
Deactivations										
DMN	mSFG	8	-6	44	52	-7.46	-	-	-	-
	mSFG	9	-4	52	44	-8.35	12	44	52	-5.28
	SFG	8	-16	34	58	-8.17	-	-	-	-
	SFG	9	-18	50	40	-4.6	16	48	42	-4.09
	MFG	8	-32	24	56	-4.91	-	-	-	-
	IPL (AG)	39	-44	-78	40	-4.59	50	-72	30	-6.82
	PCC	7/31	-4	-50	64	-4.85	4	-64	46	-5.29
	MTG	21	-54	0	-16	-4.31	-	-	-	-
	Amy	-	-	-	-	-	34	0	-24	-3.95
	Para-hipp	36	-	-	-	-	26	-22	-20	-4.5
	Hipp	-	-28	-16	-22	-6.66	30	-10	-14	-8.12
LS	TP	38	-44	12	-30	-4.79	32	18	-32	-4.84
CN	Vermis lobule I, II	-	2	-36	-18	-4.14	-	-	-	-
	Vermis lobule III	-	-2	-38	-16	-4.02	4	-38	-8	-5.01
	Cerebellum lobule IV-V	-	-6	-44	-8	-4.15	14	-42	-24	-4.49

SR, sight-reading; BA, Bormann area; NMR network for musical rhythm; SMN, sensorimotor cortex; BGN, basal ganglia network; CN, cerebellar network; ECN, executive control network; LAN, language network; SN, salience network; VN, visual network; DMN, default mode network; LS, limbic system; pre-SMA, pre-

supplementary area; PMC, premotor cortex; Thal, thalamus; MFG, middle frontal gyrus; SFG, superior frontal gyrus; IPL, inferior parietal lobule; AG, angular gyrus; MCC, middle cingulate cortex; IFG, inferior frontal gyrus; mSFG, medial superior frontal gyrus; PCC, posterior cingulate cortex; MTG, middle temporal gyrus; Amy, amygdala; Para-hipp, para-hippocampus; Hipp, hippocampus; TP, temporal pole.

Supplementary Table 2. Peak MNI coordinates of the regions exhibiting significant results in SIMP

**Results**	**Region**	**BA**	**Left Hemisphere**				**Right Hemisphere**			
			x	***y***	***z***	***t*-value**	***x***	***y***	***z***	***t*-value**
Activations										
NMR										
SMN	Pre-SMA	6	-6	12	54	11.59	12	6	62	12.39
	PMC	6	-50	-4	50	11.19	46	2	36	9.69
	Thal	-	-12	-8	8	7.2	14	-6	8	9.2
AN	STG	22	-54	-42	16	8.45	64	-20	4	10.85
	STG	41	-56	-20	4	10.84	56	-18	2	9.49
BGN	Caudate	-	-16	-2	16	8.57	18	-4	18	8.37
CN	Cerebellum lobule VI	-	-	-	-	-	36	-62	-26	12.19
ECN	SFG	10	-	-	-	-	32	52	18	8.66
	MFG	9	-	-	-	-	38	38	22	7.83
	IPL (AG)	39	-38	-46	40	9.19	-	-	-	-
	SPL	7	-	-	-	-	34	-46	42	8.26
SN	Insula	13	-46	6	2	14.36	32	20	8	12.84
	MCC	32	-	-	-	-	8	20	38	10.37
LAN	IFG	44	-44	10	6	13.58	44	18	10	13.75
	IFG	45	-40	18	4	10.56	40	26	10	12.31
VN	IOG	19	-36	-84	-10	8.31	-	-	-	-
	Lingual gyrus	18	-	-	-	-	22	-84	-8	12.89
	Calcarine cortex	18	-16	-98	-4	11.44	-	-	-	
	Fusiform gyrus	37	-34	-58	-16	7.43	-	-	-	-
Deactivations										
NMR										
SMN	M1	4	-14	-34	78	-9.02	2	-34	64	-10.93
	S1	1	-	-	-	-	24	-42	72	-9.07
	SPL	7	-20	-46	74	-8.6	-	-	-	-
DMN	SFG	9	-22	44	46	-11.5	-	-	-	-
	SFG	10	-14	64	20	-9.05	-	-	-	-
	mSFG	10	-6	64	18	-9.07	16	68	12	-8.74
	mSFG	8	-6	44	52	-8.9	-	-	-	-
	PCU	31	-2	-46	54	-10.13	2	-62	36	-10.98
	IPL	39	-54	-68	40	-6.92	56	-64	28	-9.36
	Hipp	-	-22	-12	-22	-6.09	-	-	-	-
RS	OFC	11	-2	40	-20	-7.83	2	50	-20	-7.7
LS	TP	38	-38	22	-28	-7.76	-	-	-	-

AN, auditory network; STG, superior temporal gyrus; SPL, superior parietal lobule; IOG, inferior occipital gyrus; M1, primary motor cortex; S1, primary somatosensory cortex; OFC, orbitofrontal cortex; PCU, precuneus; also refer to Supplementary Table 1 for other abbreviations.

Supplementary Table 3. Peak MNI coordinates of the regions exhibiting significant results in SR_{FIMP}

Results	Region	BA	Left Hemisphere				Right Hemisphere			
			x	*y*	*z*	*t*-value	*x*	*y*	*z*	*t*-value
Activations										
NMR	SFG	6	-	-	-	-	20	12	46	4.78
	MFG	6	-	-	-	-	34	6	50	6.82
SMN	Pre-SMA	6	-6	12	64	6.15	-	-	-	-
	PMC	6	-36	-2	46	8.98	44	2	48	6.13
CN	Cerebellum lobule VI	-	-	-	-	-	32	-70	-24	7.17
ECN	MFG (FEF)	8	-	-	-	-	30	10	46	5.37
	MFG	9	-	-	-	-	40	36	24	4.42
	SFG	10	-	-	-	-	30	42	22	6.2
	IPL	7	-32	-62	38	5.3	-	-	-	-
	SPL	7	-20	-72	48	5.25	-	-	-	-
SN	Insula	13	-32	14	4	6.46	34	20	8	6.19
	MCC	24	-6	16	26	4.35	-	-	-	-
LAN	IFG	44	-46	12	2	5.78	44	12	26	4.9
	IFG	45	-36	49	10	6.24	-	-	-	-
VN	IOG	18	-22	-92	-10	7.27	-	-	-	-
	MOG	18	-20	-98	6	9.34	-	-	-	-
	SOG	18	-12	-92	8	7.84	-	-	-	-
	Lingual gyrus	18	-12	-82	-8	6.87	20	-74	-12	8.83
	Calcarine cortex	17	-	-	-	-	14	-76	2	8.32
	Fusiform gyrus	37	-	-	-	-	28	-68	-14	7.84
Deactivations										
DMN	mSFG	8	-8	48	48	-6.79	-	-	-	-
	mSFG	9	-2	56	30	-8.16	-	-	-	-
	mSFG	10	-2	58	24	-6.78	-	-	-	-
	SFG	8	-16	46	48	-6.77	-	-	-	-
	PCU	31	-8	-54	40	-6.33	-	-	-	-
	MTG	21	-60	-4	-20	-6.37	54	0	-28	-5.19
	TP	38	-40	20	-37	-5.42	48	6	-26	-5.44
	Hipp	-	-28	-14	-22	-8.99	30	-14	-20	-10.84
	Para-hipp	-	-28	0	-28	-4.92	30	4	-28	-5.44
NMR										
SMN	SMA	6	-	-	-	-	2	-24	58	-4.38
	S1	1	-2	-32	56	-6.06	-	-	-	-
	M1	4	-14	-36	72	-5.92	2	-34	60	-8.21
ECN	SPL	7	-18	-48	76	-9.27	22	-48	74	-6.92
RS	OFC	11	0	28	-18	-8.34	-	-	-	-

FEF, frontal eye field; SOG, superior occipital gyrus; SMA, supplementary motor area; refer to Supplementary Table 1-2 for other abbreviations.

Supplementary Table 4. Peak MNI coordinates of the regions exhibiting significant results in FIMP

Results	Region	BA	Left Hemisphere				Right Hemisphere			
			x	*y*	*z*	*t*-value	*x*	*y*	*z*	*t*-value
Activations										
NMR										
SMN	M1	4	-46	16	48	13.59	-	-	-	-
	Pre-SMA	6	-8	12	50	10.49	-	-	-	-
	PMC	6	-38	-2	44	11.65	42	-12	46	8.44
	Thal	-	12	-6	4	7.77	12	-4	6	7.62
	PUT	-	-	-	-	-	22	2	10	6.17
	GP	-	-	-	-	-	16	12	4	7.77
AN	STG	22	-64	-38	10	9.44	58	-38	16	9.79
CN	Cerebellum Crus I	-	-12	-78	-22	5.52	-	-	-	-
	Cerebellum lobule VI	-	-30	-62	-24	8.68	34	-68	-26	10.74
BGN	Caudate	-	-	-	-	-	18	6	14	7.48
ECN	MFG	46/10	-36	44	8	9.89	32	50	14	8.65
	IPL	39	-	-	-	-	58	-44	20	8.6
SN	Insula	13	-38	18	2	10.19	32	20	18	10.23
	MCC	8/32	-	-	-	-	10	18	42	13.87
LAN	IFG	44	-46	6	6	11.17	44	14	6	9.34
	IFG	45	-38	18	12	9.08	-	-	-	-
VN	Lingual gyrus	18/19	-10	-80	-6	5.4	26	-54	-8	6.78
	Fusiform gyrus	37/19	-	-	-	-	24	-64	-10	6.19
Deactivations										
DMN	mSFG	9	-2	56	30	-12.4	2	56	28	-10.49
	mSFG	10	-2	60	26	-11.77	2	54	12	-9.77
	mSFG	6	-2	40	52	-8.97	-	-	-	-
	PCU	23/31	-2	-48	38	-11.19	2	-48	36	-10.54
	IPL	39	-58	-64	30	-7.09	56	-62	24	-6.09
	MTG	21	-64	-14	-18	-5.14	-	-	-	-
	Para-hipp	36	-	-	-	-	30	-14	-22	-8.85
	Hipp	-	-20	-12	-20	-8.16	-	-	-	-
LS	TP	38	-58	-2	-26	-7.77	28	8	-28	-6.93

PUT, putamen; GP, globus pallidus; also refer to Supplementary Table 1-3 for other abbreviations. *Indicate also encompassed within the BGN.
